# Genomic and Ecogenomic Characterization of *Proteus mirabilis* Bacteriophages

**DOI:** 10.3389/fmicb.2019.01783

**Published:** 2019-08-06

**Authors:** Diana R. Alves, Jonathan Nzakizwanayo, Cinzia Dedi, Chara Olympiou, Aurélie Hanin, Witold Kot, Lars Hansen, Rene Lametsch, Cormac G. M. Gahan, Pascale Schellenberger, Lesley A. Ogilvie, Brian V. Jones

**Affiliations:** ^1^School of Pharmacy and Biomolecular Sciences, University of Brighton, Brighton, United Kingdom; ^2^Blond McIndoe Research Foundation, Queen Victoria Hospital, East Grinstead, United Kingdom; ^3^Queen Victoria Hospital NHS Foundation Trust, East Grinstead, United Kingdom; ^4^Department of Biology and Biochemistry, University of Bath, Bath, United Kingdom; ^5^School of Pharmacy, Queen’s University, Belfast, United Kingdom; ^6^APC Microbiome Ireland, University College Cork, Cork, Ireland; ^7^Department of Plant and Environmental Sciences, University of Copenhagen, Copenhagen, Denmark; ^8^Department of Food Science, University of Copenhagen, Copenhagen, Denmark; ^9^School of Pharmacy, University College Cork, Cork, Ireland; ^10^School of Life Sciences, University of Sussex, Brighton, United Kingdom

**Keywords:** phage therapy, ecogenomics, biofilms, catheters, bacteriophage

## Abstract

*Proteus mirabilis* often complicates the care of catheterized patients through the formation of crystalline biofilms which block urine flow. Bacteriophage therapy has been highlighted as a promising approach to control this problem, but relatively few phages infecting *P. mirabilis* have been characterized. Here we characterize five phages capable of infecting *P. mirabilis*, including those shown to reduce biofilm formation, and provide insights regarding the wider ecological and evolutionary relationships of these phages. Transmission electron microscopy (TEM) imaging of phages vB_PmiP_RS1pmA, vB_PmiP_RS1pmB, vB_PmiP_RS3pmA, and vB_PmiP_RS8pmA showed that all share morphologies characteristic of the *Podoviridae* family. The genome sequences of vB_PmiP_RS1pmA, vB_PmiP_RS1pmB, and vB_PmiP_RS3pmA showed these are species of the same phage differing only by point mutations, and are closely related to vB_PmiP_RS8pmA. Podophages characterized in this study were also found to share similarity in genome architecture and composition to other previously described *P. mirabilis* podophages (PM16 and PM75). In contrast, vB_PimP_RS51pmB showed morphology characteristic of the *Myoviridae* family, with no notable similarity to other phage genomes examined. Ecogenomic profiling of all phages revealed no association with human urinary tract viromes, but sequences similar to vB_PimP_RS51pmB were found within human gut, and human oral microbiomes. Investigation of wider host-phage evolutionary relationships through tetranucleotide profiling of phage genomes and bacterial chromosomes, indicated vB_PimP_RS51pmB has a relatively recent association with *Morganella morganii* and other non-*Proteus* members of the *Morganellaceae* family. Subsequent host range assays confirmed vB_PimP_RS51pmB can infect *M. morganii*.

## Introduction

The opportunistic pathogen *Proteus mirabilis* is a common cause of catheter-associated urinary tract infections (CAUTIs) and can significantly complicate the care of patients undergoing long-term urethral catheterization (Mobley, 1996; Stickler, 2008, 2014). *P. mirabilis* infection often leads to encrustation and blockage of catheters in these individuals, which if unnoticed, can result in the reflux of infected urine to the upper urinary tract and serious clinical symptoms including pyelonephritis, septicaemia, and shock (Mobley, 1996; Jacobsen et al., 2008; Stickler, 2008, 2014). Although blockage is usually not a problem in hospitalized patients, the majority of long-term catheterized individuals are cared for in the community where continual clinical surveillance is not possible, and blockage is often not detected until subsequent symptomatic complications arise (Stickler, 2014). As a result, catheter blockage is the underlying cause of a large proportion of emergency hospital referrals for individuals undergoing long-term catheterization (Kohler-Ockmore and Feneley, 1996; Stickler, 2014).

The blockage of catheters by *P. mirabilis* is derived from the ability of this organism to form dense biofilms on the surface of catheters, in conjunction with the production of a potent urease enzyme (Griffith et al., 1976; Jones and Mobley, 1987; Stickler et al., 1993; Jones et al., 2005). This highly active enzyme hydrolyses urea present in the urine and generates ammonia, which in turn elevates urinary pH (Hedelin et al., 1984; Cox and Hukins, 1989; Stickler et al., 1993; Holling et al., 2014a,b). Under these alkaline conditions, calcium and magnesium phosphates precipitate to form microcrystalline aggregates suspended in the urine (Stickler et al., 1993; Stickler, 2008). As the biofilm continues to develop, these crystals become trapped in the growing community, where the exopolymeric matrix further stabilizes and enhances their growth, eventually leading to the development of a mineralized crystalline biofilm structure that can block urine flow (Stickler et al., 1993; Stickler, 2008).

Although catheters designed to prevent CAUTIs and impede biofilm formation have been developed, they remain prone to encrustation and blockage by *P. mirabilis*, and are considered ineffective in long-term settings (Morris et al., 1997; Morgan et al., 2009; Stickler, 2014). This includes catheters approved for use within the United Kingdom National Health Service; for example, antimicrobial catheters coated with silver or nitrofurazone have been shown to have no significant impact on the incidence of CAUTI, even during short-term catheterization (Pickard et al., 2012). *P. mirabilis* also frequently causes chronic infection in long-term catheterized patients and can persist after catheter changes and multiple rounds of standard antibiotic treatment (Kunin, 1997). Therefore, there are currently no fully effective approaches to prevent or manage catheter blockage due to *P. mirabilis* CAUTI, and new strategies are required to deal with this persistent and growing clinical problem.

Previously, we have explored the use of bacteriophage (or phage) to combat *P. mirabilis* crystalline biofilm formation, and demonstrated the potential for this approach to control catheter encrustation (Nzakizwanayo et al., 2016). A combination of three phages that infect *P. mirabilis* were applied to *in vitro* models of the catheterized urinary tract. This cocktail was able to significantly inhibit crystalline biofilm formation in experiments simulating an established infection and completely eradicated *P. mirabilis* from models in experiments simulating the early stages of infection (Nzakizwanayo et al., 2016). Other *P. mirabilis* phages have also been characterized with therapeutic applications in mind, and include the podophages PM16, PM75 and vB_PmiP_5460 (Melo et al., 2016; Morozova et al., 2016, 2018), the Siphophage pPM_01 (Wirjon et al., 2016), and the Myophage vB_PmiP_5461 (Melo et al., 2016). Notably, phages vB_PmiP_5460 and vB_PmiP_5461 have also been demonstrated to reduce crystalline biofilm formation in models of CAUTI (Melo et al., 2016).

However, there is presently a general paucity of data regarding phages that infect *P. mirabilis*, and in order to fully explore the potential for phage therapy to control this pathogen, as well as to optimize the development of effective phage cocktails, a greater understanding of phages infecting this species is required. Here we describe the full genomic sequence and characterization of phages infecting *P. mirabilis*, including those previously shown to have potential utility in controlling crystalline biofilm formation (Nzakizwanayo et al., 2016). We demonstrate how genomic and ecogenomic profiling of these phages can identify broader affiliations with various microbial ecosystems and other bacterial host species. Collectively, these genomic and ecogenomic approaches will aid the development of phage cocktails for the control of *P. mirabilis* and other urinary tract pathogens.

## Materials and Methods

### Bacterial Strains, Media, and Routine Culture

Clinical isolates of *P. mirabilis* used in this study were obtained from the Royal Sussex County Hospital (Brighton, United Kingdom), and all were isolated from urinary tract infection sites (Supplementary Table S1). All chemicals, reagents and growth media were obtained from either Thermo Fisher Scientific, United Kingdom, Oxoid, United Kingdom, or Sigma, United Kingdom, unless otherwise stated. Bacteria were routinely cultured in Luria-Bertani (LB) medium (5 g/L yeast extract, 10 g/L tryptone, 10 g/L sodium chloride) at 37°C with shaking, or on LB solidified by addition of 15 g/L Technical agar (LA). Soft agar overlays, used for phage enrichments, purification and enumeration, were derived from LB, and contained 6.5 g/L Technical agar (S-LA).

### Phage Isolation and Purification

Phages were isolated from wastewater collected from treatment plants in the United Kingdom (Anglian Water, Milton Keynes, Luton, and Sharnbrook). Enrichments for phages active against *P. mirabilis* were performed by mixing 100 mL of wastewater with 387.5 mL of LB, and subsequent inoculation with 2.5 mL of an overnight culture of *P. mirabilis* (see Supplementary Table S1 for host strains). This was incubated at 37°C overnight without shaking. 10 mL aliquots were centrifuged (3000×*g* for 30 min), and supernatants filtered into fresh sterile tubes using 0.45 μm pore syringe filters (Sartorius, United Kingdom). 3 mL S-LA inoculated with 100 μL of an overnight *P. mirabilis* culture was mixed with 100 μL of filtered enrichment, swirled gently, and immediately poured over the surface of a LA plate. Plates were incubated at 37°C for 18–20 h, and phage with the ability to infect the host strain were identified by observing zones of lysis (plaques) in the agar. To isolate distinct phage, individual plaques were picked off using Pasteur pipettes and re-suspended in 300 μL suspension medium (SM) buffer (100 mM NaCl, 10 mM MgSO4.7H_2_O, and 50 mM Tris–HCL pH 7.5, 0.01% gelatine). The resulting phage suspensions were serially diluted in SM Buffer, and the dilutions used to prepare agar overlays with overnight cultures of *P. mirabilis*, and plates incubated at 37°C overnight. To ensure phage purity, this process was repeated a further 5 times until bacterial lawns showed homogeneity of plaque morphology. An individual plaque was then picked off and re-suspended in SM buffer for use in subsequent experiments. Purified phage suspensions were stored at 4°C until required.

### Preparation of High Titre Phage Stocks

To prepare high titre stocks of isolated phage (10^10^ pfu/ml), phages were propagated on their original host strain in LB broth (Supplementary Table S1), and 100 μL of the resulting phage lysate mixed with 100 μL of a fresh overnight culture of *P. mirabilis*. This was incubated for 5 min at room temperature without shaking, before being used to prepare soft agar overlays on LB agar plates, as described above. After an overnight incubation at 37°C, plates displaying confluent lysis were selected and flooded with 3 mL of SM buffer supplemented with 2% (v/v) chloroform, before incubation at 37°C for 4 h. The resulting phage suspension was removed from the plates, centrifuged (11,000 × *g* for 10 min) to remove cell debris, and filtered using 0.22 μm pore size syringe filters. In order to further concentrate the phage, one volume of 20% PEG-8000 solution supplemented with 1M NaCl (Sigma, United Kingdom) was added to the phage suspension and incubated at 4°C overnight, before centrifugation at 11,000 × *g* for 20 min. The resulting pellet was re-suspended in SM buffer and a 1/5 volume of chloroform was added, vortexed for 30 s to mix, followed by slow centrifugation (3,000 × *g* for 15 min at 4°C). The upper aqueous phase was removed and stored at 4°C until required.

### Infection of Non-*Proteus* Species

The ability of phage to infect and replicate in alternate host species, was evaluated using spot tests of phage suspensions on agar overlays seeded with potential host bacteria. Isolates of species tested were obtained from DSMZ, and are described in Supplementary Table S1. For each bacterial species tested, 100 μL of an overnight culture was mixed with 3 mL of S-LA and poured onto a LA plate. Plates were left to dry for 20 min at 37°C. Phage lysates were standardized to a titre of 10^9^ PFU/mL, and 10 μL of each lysate was spotted onto the bacterial lawns at concentrations ranging from 10^3^ to 10^9^ PFU/mL, and plates incubated overnight at 37°C. This assay was performed in triplicate, and evidence of phage replication taken as formation of plaques in the region of the spotted phage suspension.

### Electron Microscopy

Purified phage particles at a concentration of 10^9^ PFU/mL were deposited on 300 mesh carbon-coated copper grids (Agar Scientific, United Kingdom) and negatively stained with 1% uranyl acetate (pH 4) as follows: the surface of the copper grids were ionized for 2 min immediately prior to sample deposition. 5 μL of phage lysate were spotted on the surface of the grid and allowed to stand for 1 min. This was followed by 30 sec negative staining and subsequent air drying of the grid. Visualization was performed using a JEOL JEM-1400Plus TEM, operated at 120 kV (pixel size = 0.1 nm), equipped with a Gatan OneView 4K camera with automatic drift correction.

### Phage DNA Sequencing, Assembly, and Inference of Physical Genome Structure

Phage genomic DNA was extracted through a direct plaque sequencing method described by Kot et al. (2014). DNA sequencing libraries were prepared using the Nextera XT DNA kit (Illumina, San Diego, CA, United States), according to the manufacturer’s protocol. Individually tagged libraries were sequenced as part of a flow cell as 2 × 250 base paired-end reads using the Illumina MiSeq platform (Illumina, San Diego, CA, United States). Reads were analyzed, trimmed, and assembled using CLC Genomic Workbench version 6.5.1, as described previously (Kot et al., 2014).

Nanopore sequencing was conducted using multiplex barcoded sequencing of phage genomic libraries on the Oxford Nanopore Technologies (ONT) MinION sequencing platform. Genomic DNA was extracted from high titre phage stocks (10^10^ pfu/mL) using the Zinc precipitation method originally described by Santos (1991), with some minor modifications previously outlined in Ogilvie et al. (2012). The quantity and purity of the resulting DNA was tested using the Qubit 2.0 and the Nanodrop 2000, respectively. Sequencing libraries were prepared according to the ONT protocols for the Native Barcoding Kit (EXP-NBD103), and the Ligation sequencing Kit 1D (SQK-LSK108). For each phage, 1 μg of total genomic DNA was treated with the NEBNext End repair/dA-tailing Module (E7546; New England Biolabs), purified using 1× volume AMPure XP beads (Beckman Coulter), then eluted in nuclease – free water (25 μL). Next, 500 ng of each end – repaired adenylated DNA was ligated to one of the Native Barcodes (selected from NB01 to NB12; ONT) using the Blunt/TA Master Mix (M0367; New England Biolabs), cleaned using the AMPure XP beads, then eluted in nuclease – free water (26 μL). The barcoded samples were combined in equimolar amounts to produce a pooled sample of 700 ng in 50 μL nuclease-free water and Barcode Adapter ligation performed using T4 DNA ligase in the NEBNext Quick Ligation Module (E6056; New England Biolabs). The resulting library was eluted in the Nanopore Elution Buffer (15 μL) and amount of DNA quantified using the Qubit 2.0 to confirm total DNA recovery of ∼500 ng. The final library (12 μL) was mixed with Nanopore Running Buffer with Fuel Mix (35 μL), Library Leading Beads (25.5 μL), and Nuclease-fee water (2.5 μL). This mixture was loaded onto a pre-primed single R9.4/FLO-MIN106 flow cell in a MinION Mk1B, and run for 48 h.

The resulting MinION reads were subject to basecalling using Albacore v2.3.1, and reads demultiplexed and trimmed using Porechop (v0.2.4 October 2018^1^). Nanopore reads with a minimum length of 500 nucleotides were subsequently assembled using CANU 1.17 (Koren et al., 2017) using default settings for assembly of raw nanopore reads, and assuming a maximum possible genome size of 50 or 100 kb. MinION assemblies were used to infer the physical structure and terminal repeat sequences of phage genomes through Mauve alignments v2.3.1 (Darling et al., 2004; implemented in Geneious 9.1.8), with initial Illumina assemblies. Terminal repeats in podophage genomes were detected using the Geneious 9.1.8 repeat finder tool as well as through comparison to the previously described podophage PM16 and PM75 (Morozova et al., 2016, 2018). Subsequently, Illumina assemblies were corrected manually to reflect the predicted physical genome structure of phage, and verified by comparison of predicted vs. actual restriction profiles of the phage genomes.

### Restriction Digest of Phage Genomes

For restriction digest of phage genomes, phages were propagated on host strains and purified and harvested by filtration (0.22 μm pore size), followed by concentration using VIVASPIN^®^ 15 10,000MWCO PES membranes (Sartorius). Concentrated phage lysate (1 mL) was treated with 10 μg DNAse I (Sigma-Aldrich) and 5 μg RNase A (Sigma-Aldrich) for 30 min at 37°C, prior to extraction of phage DNA using the QIAmp Min Elute Virus Spin kit (QIAGEN). Recovered phage DNA was digested using EcoRI and XbaI individually, according to manufacturer’s instructions (Fermentas Fast Digest^®^ enzymes), and restriction fragments separated using agarose gel electrophoresis.

### Annotation and Analysis of Phage Genomes

Open reading frames (ORFs) were predicted from Illumina assemblies using Glimmer V3 (implemented in Geneious 9.1.8). The putative functions of the ORFs were predicted using translated ORF amino acid sequences in BlastP searches of the full non-redundant protein sequence database and NCBI CD-Search queries of the Conserved Domain Database (encompassing models from COG, Pfam, SMART, PRK, and TIGRFAM databases). Only BlastP hits with a minimum 20% identity and e-values of 1e^–5^, and Conserved Domain hits with e-values on 1e^–2^ were used to assigned putative functions to ORFs. Phage encoded ORFs were also used to search the Virulence Factor Database (October 2017) (Chen et al., 2005, 2016), and the MEGARes antibiotic resistance gene database (December 2016) (Lakin et al., 2017). In both cases, only hits generating a minimum of 20% identity and e-values of 1e^–5^ or lower were considered valid.

### Analysis of Proteins in Mature Phage Particles

The phage proteins were identified as previously described in Carstens et al. (2016). Briefly, 75 μL of the purified phage solution was mixed with 75 μL of 1% SDS and incubated for 30 min at 80°C followed by Trichloroacetic acid (TCA) precipitation. The proteins were re-solubilized in 8 M urea, 45 mM dithiothreitol (DTT), and 50 mM Tris, pH 8.0, reduced and alkylated. The proteins were trypsin digested and the resulting peptides analyzed using a Dionex 3000 rapid separation liquid chromotography (RSLC) ultra high performance LC (UHPLC) system (Thermo Fisher Scientific, Hvidovre, Denmark) with an Aeris PEPTIDE 1.7 μm XB-C18, 150 mm × 2.1 mm column (Phenomenex, Værløse, Denmark) coupled to a Q Exactive mass spectrometer (Thermo Fisher Scientific, Hvidovre, Denmark). The resulting data were analyzed with Proteome Discoverer software (version 1.4, Thermo Fisher Scientific, Hvidovre, Denmark), using an in house protein database based on the obtained DNA sequences. The search results were filtered using the integrated Target decoy peptide–spectrum matches (PSM) validator algorithm set to a q-value of <0.01, which ensures a peptide-spectrum match false discovery rate less than 0.01.

### Analysis of Tetranucleotide Usage Profiles in Phage Genomes

Tetranucleotide usage profiles from phage genomes and bacterial chromosomes were calculated using the method of Teeling et al. (2004), adapted by Ogilvie et al. (2012, 2013). Sequences were extended by their reverse complement before usage profiles for all possible 256 tetranucleotide combinations were calculated over a 1 nt sliding window. The resulting counts for each tetranucleotide were converted to *Z*-scores and used for further analyses with the R packages Vegan and BioDist. For analysis of similarity (ANOSIM), *Z*-scores were utilized without further processing, whereas for non-metric multidimensional scaling (nMDS) *Z*-scores from each sequence were first used to construct Euclidian distance matrices using Vegan. The resulting matices were then used for nMDS ordination with 1000 random starts. Cladograms were constructed from Pearson dissimilarity matrices of *Z*-scores (generated using the BioDist package), using the neighbor-joining algorithm in Vegan (with 500 bootstrap replicates). The resulting cladograms were further analyzed to derive the majority consensus tree, and annotated using Dendroscope v3.5.7.

### Representation of Phage-Encoded Sequences in Metagenomic Datasets

Ecogenomic analysis of sequences with similarity to ORFs encoded by *P. mirabilis* phage in metagenomic datasets was performed as previously described (Ogilvie et al., 2012, 2013). Datasets searched represent a range of different habitats within or on the human body, as well as range of habitats from marine and terrestrial environments. The details of metagenomic datasets searched can be found in Supplementary Table S2. Datasets were searched using tBlastn with amino acid sequences from each predicted phage ORF, and valid hits were considered to be those generating ≥35% identity over ≥50% of the query sequence and an e-value of ≤1e^–5^. Valid hits were subsequently used to calculate the relative abundance of each phage-encoded ORF in each dataset (expressed as Hits/Mb of sequence data). The cumulative relative abundance of ORFs encoded by each phage was taken as the sum of all individual ORF relative abundances. Blast searches and calculation of relative abundance were automated using a custom PERL script as described in Ogilvie et al. (2018) (access and support is available on request to authors), which implemented BLAST v2.2.29 with default settings.

### Statistical Analysis

All statistical analyses were performed using Prism 6.0c for Mac OS X (GraphPad Software, Inc., United States), or the R packages Vegan and BioDist (for nMDS, ANOSIM, and Cladograms). Significant differences in cumulative relative abundances between metagenomes were assessed using the Kruskal-Wallis test with Dunn’s correction for multiple comparisons.

### Phage Names and Accession Numbers

All phage sequences were named according to guidelines from the International Committee on Viral Taxonomy (Adriaenssens and Brister, 2017), and have been deposited in GenBank under the following accession numbers: Proteus_phage_ vB_PmiP_RS1pmA (also representing RS1pmB and RS3pmA) – MG575418; Proteus_phage_vB_PmiP_RS8pmA – MG575419; Proteus_phage_vB_PmiM_RS51pmB – MG575421.

## Results

### Phenotypic Characterization of *P. mirabilis* Phage

Phages capable of infecting *P. mirabilis* (designated Proteus_ phage_vB_PmiP_RS8pmA, and Proteus_phage_vB_PmiM_ RS51pmB) were isolated from sewage using clinical isolates of *P. mirabilis* recovered from urinary tract infections. Phages were initially characterized based on plaque and virion morphology and compared to previously isolated phages (Proteus_phage_vB_PmiP_RS1pmA, RS1pmB, and RS3pmA), which have been shown to reduce *P. mirabilis* crystalline biofilm formation when tested *in vitro* infection models (Nzakizwanayo et al., 2016). For simplicity, we refer to phage by their unique identifiers for the remainder of the manuscript: RS1pmA, RS1pmB, RS3pmA, RS8pmA, and RS51pmB.

**FIGURE 1:**
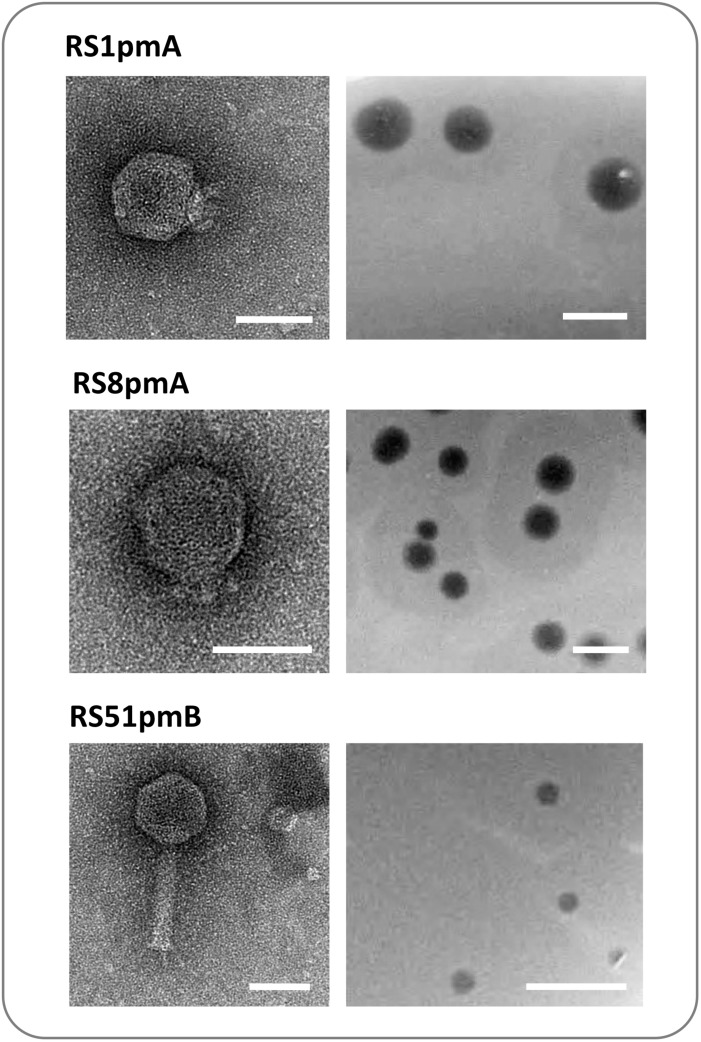
Capsid and plaque morphology of *Proteus mirabilis* phages. Images show transmission electron microscopy (TEM) projections of negatively stained phage (left), and examples of plaques formed by each phage on lawns of host bacteria (right). Phage capsid structures were congruent with membership of the *Podoviridae* family (RS1pmA and RS8pmB), and the *Myoviridae* family (RS51pmB). RS1pmA is used to represent the previously described group of phages RS1pmA, RS1pmB and RS3pmA, which all have analogous capsid morphology (Nzakizwanayo et al., 2016). Scale bars show 50 nm on TEM images and 1 cm on images of plaques. TEM images shown are representative of at least 10 fields of view containing one or more virions.

Analysis of virion structure by TEM (Figure 1) showed that RS8pmA is likely a member of the *Podoviridae* family based on the short length of tails (∼10 nm in length), and similar to the previously isolated podophage RS1pmA, RS1pmB, and RS3pmA (Nzakizwanayo et al., 2016). RS8pmA was also observed to generate plaques with similar morphology to the previously isolated phages, including surrounding halos that are considered indicative of polysaccharide depolymerase (PD) activity (Figure 1; Sutherland et al., 2004). In contrast, TEM showed RS51pmB virion morphology aligned with membership of the *Myoviridae* family (Figure 1). RS51pmB generated small circular plaques of a consistent diameter, with no evidence of PD activity (Figure 1). All phages generated clear plaques with no indication of lysogenic replicaiton apparent.

### Analysis of *P. mirabilis* Phage Genomes and Inference of Physical Structure

**FIGURE 2:**
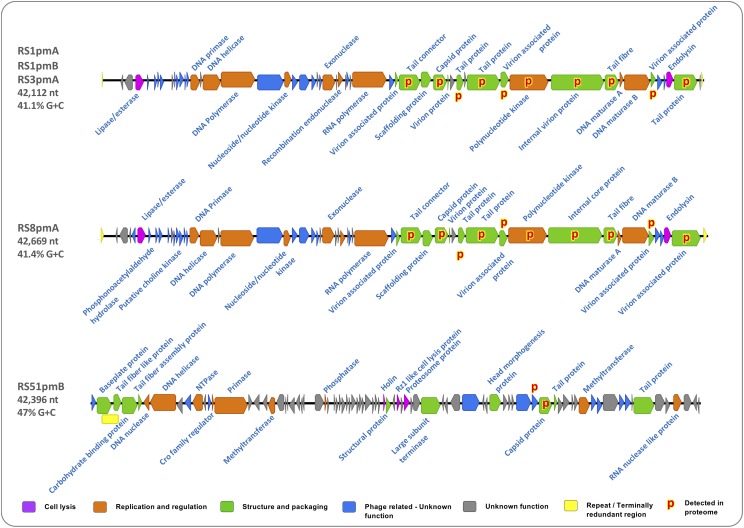
Functional content and characteristics of *P. mirabilis* phage genomes. Maps show the physical gene architecture of *P. mirabilis* phages RS1pmA, RS51pmB, and RS8pmA. Block arrows represent positions of open reading frames (ORFs) with colors indicating affiliation to broad functional groups relevant to phage replication, as denoted by the associated key: Cell Lysis, predicted to encode for activities involved in lysis of host cells; Replication and Regulation, predicted to encode for activities involved in replication of phage genomes and regulation of this process; Structure and Packaging, predicted to encode components of the phage capsid or support packaging of new phage genomes during replication; Unknown Phage related, unknown function but homologous sequences identified in other phage genomes; Unknown, generated no valid hits in BlastP or Conserved Domain searches. Putative functions of each ORF were predicted using BlastP and conserved domains (CD) searches against the nr database (BlastP: minimum 20% identity, 1e^– 5^ or lower; CD: 1e^– 2^ or lower).

Phage genomes were initially generated using Illumina sequencing to an average depth of ∼181× coverage. However, the repeat sequences that are often present at the terminal ends of podophage genomes, and other terminally redundant features of phage genomes (arising from particular replication strategies), can lead to mis-assembly of short read data. To account for this we also generated phage genome assemblies from long-read nanopore sequencing methods, which were used in combination with restriction digests to infer the correct physical structure of phage genomes, identify terminal repeats, and correct Illumina assemblies (Figure 2).

This analysis indicated that the initial Illumina assemblies of phage genomes generated in this study (RS1pmA, RS1pmB, RS3pmA, RS8pmA, and RS51pmB) did not reflect the correct physical structure of phage genomes, most likely due to merging of terminal repeat regions during assembly. Restriction digest of phage genomes confirmed the nanopore-corrected Illumina assemblies provided a more accurate representation of the physical genome structure of these phages (Supplementary Figures S1, S2). In the case of RS51pmB, MinION data, and restriction digests also indicated this phage genome to be circularly permuted with terminal redundancies, indicative of the headful packaging mechanism observed in other members of the *Myoviridiae* (Schwudke et al., 2008; Casjens and Gilcrease, 2009; Yap and Rossmann, 2014). However, the packaging strategies used by these phages, the exact sequence and positions of repeats or regions of terminal redundancy, and the related molecular details of genome replication will require further detailed studies to confirm and elucidate.

Genome sequences of RS1pmA, RS1pmB, and RS3pmA revealed these to be species of the same phage, differing only by 7 point mutations at several locations in their genome sequences (Figure 2 and Supplementary Figure S3). Five of these point mutations were predicted to lead to amino acid substitutions, and were predominantly associated with genes predicted to encode structural proteins in mature viral particles, including those likely to play a direct role in host attachment such as tail fiber proteins (Supplementary Figure S2). Due to the high level of identity between RS1pmA, RS1pmB and RS3pmA, subsequent analyses were conducted using RS1pmA as a representative of this group of phages. RS8pmA also exhibited notable similarities in genome sequence and gene synteny to the RS1 group of phages, in keeping with these phages all exhibiting similar virion morphology and belonging to the *Podoviridiae* family (Figure 2, Supplementary Figure S4, and Supplementary Data S1). In contrast, RS51pmB was found to encode distinct genome organization and gene contents from the other phages analyzed, congruent with differences in the virion morphology of this phage as compared to RS1pmA and RS8pmA (Figure 2 and Supplementary Data S1).

When ORFs in each genome were assigned to broad categories based on putative function (as described in Figure 2), similar proportions of genes predicted to be involved in virion structure, aspects of regulation and replication, and host cell lysis, were observed in most phage genomes (Figure 2). As with many other phage genomes, a high proportion of ORFs with no identifiable function were also observed in all phage genomes. Although some of the ORFs with unknown function were found to also have homologs in other phage genomes, many showed no similarities to any sequences currently present in the NCBI non-redundant database (Figure 2). None of the phage genomes characterized here encoded identifiable genes for virulence factors or antibiotic resistance determinants.

### Analysis of Mature Virion Proteomes

To support *in silico* predictions of virion-associated structural genes, the proteome in mature phage particles was also analyzed (Figure 2 and Supplementary Table S3). In RS1pmA and RS8pmA this confirmed predictions of virion-associated genes based on BlastP and Conserved Domain homologies (Figure 2 and Supplementary Table S3). However, for RS51pmB, proteomic analysis failed to detect the predicted products for most capsid-associated ORFs (Figure 2 and Supplementary Table S3). There were also indications that proteins related to genes of unknown function (but with homologs in other phage genomes; RS51pmB), or ORFs predicted to encode polynucleotide kinase (RS1pmB and RS8pmA), are potentially present in mature virions (Figure 2 and Supplementary Table S3). Alternatively, the detection of proteins assigned functions in replication and regulation could conceivably result from protein carry over in phage lysates, and the role, if any, of these proteins in the mature virion will require further investigation to confirm.

### Representation of *P. mirabilis* Phage Genes in Other Phage Genomes

**FIGURE 3:**
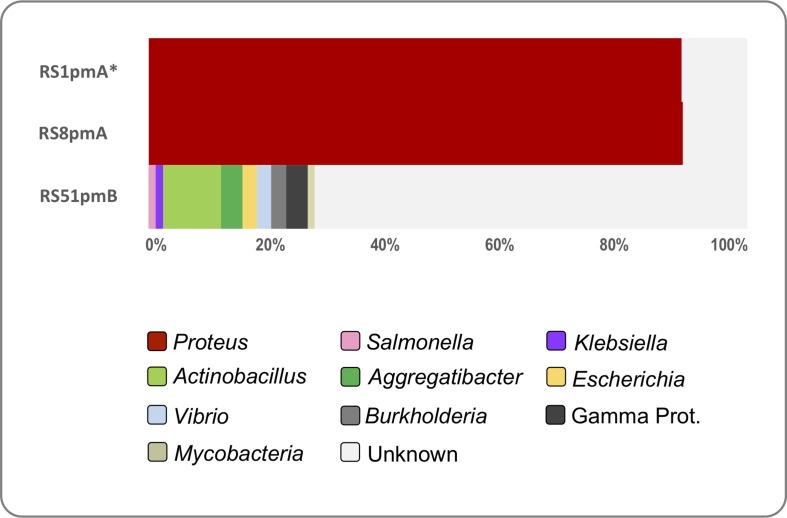
Representation of *P. mirabilis* phage-encoded ORFs in other phage genomes. The representation of *P. mirabilis* ORF homologs in other phage genomes was explored using tBLASTn searches of 715 complete phage genomes. The proportion of ORFs affiliated to other phage genomes is based on top hit by bit score (min 35% identity, over ≥25 amino acids, 1e^– 5^ or lower). Hits were categorized by genus of host bacterial species for phage genomes generating each hit. Legends associated with charts describe bacterial genera represented. The “Gamma Proteobacteria” group included *Hamiltonia* sp., *Listonella* sp., and *Pseudoalteromonas* sp., which were each represented by less than two hits across all phage genomes. RS51pmB also only generated a single hit to phage infecting *Mycobacterial* sp. ORFs generating no valid hits in tBlastn searches are designated as “Unknown.” ^*^RS1pmA is used to represent the previously described group of phages RS1pmA, RS1pmB, and RS3pmA (Nzakizwanayo et al., 2016), which were found to differ only by point mutations (see Supplementary Figure S3).

To evaluate similarities between the *P. mirabilis* phages isolated here and phage infecting other bacterial host species, we used all ORFs encoded by each *P. mirabilis* phage to search a collection of 715 publicly available phage genomes. This analysis demonstrated that the podophages RS1pmA and RS8pmA exhibit high levels of similarity to other available *Proteus* phage genomes, with 89–90% of ORFs encoded by these phages affiliated predominantly with PM16 (NC_027342), and PM75 (NC_027363) (Morozova et al., 2016, 2018; Figure 3 and Supplementary Data S1). The similarity of RS1pmA and RS8pmA was explored further through pairwise alignments of these phage genomes with complete genomes of PM16 and PM75. This confirmed overall similarity and gene synteny between these phages (as well as providing further support for validity of the MinION-corrected genome structures), but also highlighted distinct variations across the genome sequences (Supplementary Figure S5). In contrast, RS51pmB did not exhibit any notable similarity to other *Proteus* phage genomes, and the majority of RS51pmB ORFs were not affiliated with any phage genomes searched using this approach (72.3%). The RS51pmB ORFs that could be affiliated with other phage genomes were associated with phage infecting a diverse array of Proteobacterial hosts, predominantly Gamma-proteobacteria, including *Salmonella, Klebsiella, Escherichia*, *Vibrio*, *Aggregatibacter* and *Actinobacillus* spp. (Figure 3 and Supplementary Data S1).

### Evaluation of Broader Evolutionary Host-Phage Relationships

To gain further insight into the potential broader evolutionary relationships of the *P. mirabilis* phages isolated in this study, we used alignment-free correlations of tetranucleotide usage patterns to discern potential associations between *Proteus* phage genomes characterized within the present study, and bacterial chromosomes from a range of species. This approach exploits similarities in global nucleotide usage patterns that develop between phage genomes and the chromosomes of long-term bacterial hosts (Pride et al., 2006; Ogilvie et al., 2012, 2013). The underlying hypothesis is that in phage which have recently become capable of infecting and replicating in a new host species, nucleotide usage profiles adapt more slowly and initially still reflect previous long-term hosts (Ogilvie et al., 2013, 2018).

**FIGURE 4:**
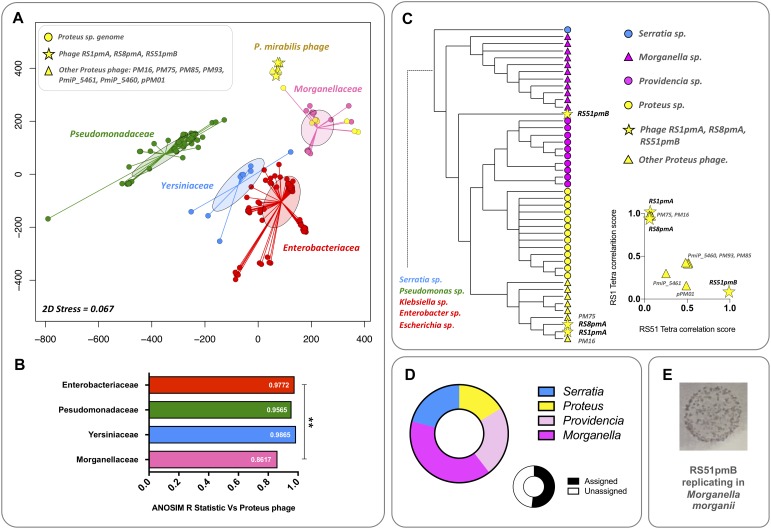
Evaluation of broader evolutionary relationships and host affiliations. The potential for broader evolutionary relationships between *P. mirabilis* phages and other bacterial hosts were explored by comparing *P. mirabilis* phages with bacterial genomes belonging to genera containing the most common Gram-negative pathogens of the catheterized urinary tract (*Proteus, Providencia, Morganella, Pseudomonas, Escherichia, Klebsiella, Enterobacteria*, and *Serratia*) (Stickler, 2014). **(A)** Unsupervised ordination using nMDS was initially used to visualize relationships between all sequences based on tetranucleotide usage profiles (Ogilvie et al., 2012, 2013). Bacterial genomes were classified by family. For nMDS, points show position of individual genomes in the final ordination, with connecting lines indicating relationship to the group centroid. Filled ellipses show standard deviation of group dispersion relative to the group centroid. Ordination were based on an Euclidean distance matrix of tetranucleotide usage profiles from each sequence. nMDS analysis was conducted with 1000 random starts using the R package Vegan. **(B)** ANOSIM between groups of sequences represented in the nMDS ordination. Charts show the ANOSIM R Statistic for each group of bacterial genomes vs. the *P. mirabilis* phage group, where an increasing strength of separation between groups is indicated as the R Statistic approaches 1. ^∗∗^Denotes statistical significance of R Statistics between groups, *P* ≤ 0.001 (calculated as part of the ANOSIM in the R Package Vegan). **(C)** Cladogram based on tetranucleotide usage profiles showing relationship between *P. mirabilis* phage sequences and genomes from *Morganellaceae* and *Yersiniaceae* groups represented in nMDS ordinations. The cladograms were constructed from a Pearson dissimilarity matrix of tetranucleotide profiles from all sequences used in nMDS analyses as majority consensus trees from 500 bootstrap replicates (as calculated by the R package BioDist). The cladogram presented shows a sub-region of the larger tree populated by *P. mirabilis* phages and the most closely related bacterial genomes by this analysis. *Chart Inset:* shows differences between *P. mirabilis* phage sequences, based on scatter plots of phage RS1pmA and RS51pmB tetranucleotide correlation scores with other phage sequences. **(D)** To further test relationships in phage RS51pmB indicated by tetranucleotide analyses, ORFs from this phage were searched against all sequences from genus level taxonomic groups represented in cladograms (*Proteus, Providencia, Morganella*, and *Serratia*) using BlastP, and hits used to assign a taxonomic affiliation. Only hits with a maximum e-value of 1e^– 3^ were considered valid. Charts show the proportion of assignable ORFs affiliated to each genus, and the proportion of assigned and unassigned ORFs. **(E)** To test if the relationships with non-*Proteus* species indicated in tetranucleotide and Blast analyses were relevant to the host range of this phage, the ability of RS51pmB to replicate in species other than *P. mirabilis* was tested (Supplementary Table S1). The image presented demonstrates the ability of phage RS51pmB to use *M. morganii* as an alternate host species (Supplementary Table S1). Host range assays were performed in triplicate at phage titres ranging from ∼10 to 10^7^ PFU, with the image shown representative of *M. morganii* lawns exposed to 10^7^ PFU of RS51pmB.

Initial relationships were explored between the phages characterized in this study, other available *P. mirabilis* phages, and a collection of Gammaproteobacteria genomes representing genera to which other common urinary tract pathogens belong (Stickler, 2014). Initially, unsupervised ordination of phage and bacterial genomes by nMDS coupled with ANOSIM, was used to visualize potential relationships between *Proteus* phages and bacteria from different families (Figures 4A,B). This analysis indicated no detectable relationship between *Proteus* phages and members of the *Enterobacteriacea*, *Pseudomonadaceae*, and *Yersiniaceae* represented (Figures 4A,B). As expected, the *Morganellaceae* group, to which *Proteus* sp. belong, showed the closest association with *Proteus* phages (Figures 4A,B).

To explore these potential relationships in more detail, phage sequences and bacterial genomes were used to construct cladograms based on tetranucleotide profiles (Figure 4C). While this supported the nMDS and ANOSIM analyses and showed a general association of *Proteus* phages with bacterial genomes from species belonging to the *Morganellaceae*, this also revealed distinct differences between RS51pmB and other phages. RS51pmB did not cluster with other *Proteus* phages and was less closely associated with *P. mirabilis* genomes (Figure 4C). This separation was also observed directly in tetranuclotide usage profiles of RS1pmA and RS51pmB, when these were correlated with other *Proteus* phages and used to construct scatterplots (Figure 4C inset). To further test this observation, we also used Blast searches to affiliate each ORF encoded by RS51pmB with all bacterial genomes belonging to genera clustering with these phages in cladograms (*Proteus, Morganella, Providencia*, and *Serratia*) (Figure 4D). This also supported an association of RS51pmB with non-*Proteus* species, with the majority of assignable ORFs being affiliated to *Morganella* and *Providencia* sp. (Figure 4D).

Collectively, these analyses indicated the potential for RS51pmB to have a previous or ongoing evolutionary relationship with non-*Proteus* host species from these bacterial Genera. To explore this further, we tested the potential for RS1pmA, RS8pmA, and RS51pmB to replicate in potential alternate hosts represented in relevant clusters of the cladogram. Although it was not possible to use an exhaustive panel of species and strains, these experiments did demonstrate RS51pmB to be capable of infecting *Morganella morganii* (albeit inefficiently compared to *P. mirabilis*), supporting the inference of a relationship with non-*Proteus* hosts based on tetranucleotide usage profiles (Figure 4E). However, it should be noted that more extensive host-range assays will be required to determine how robust these relationships are, and fully understand the host range of these phages.

### Ecogenomic Profiling of *P. mirabilis* Phage

**FIGURE 5:**
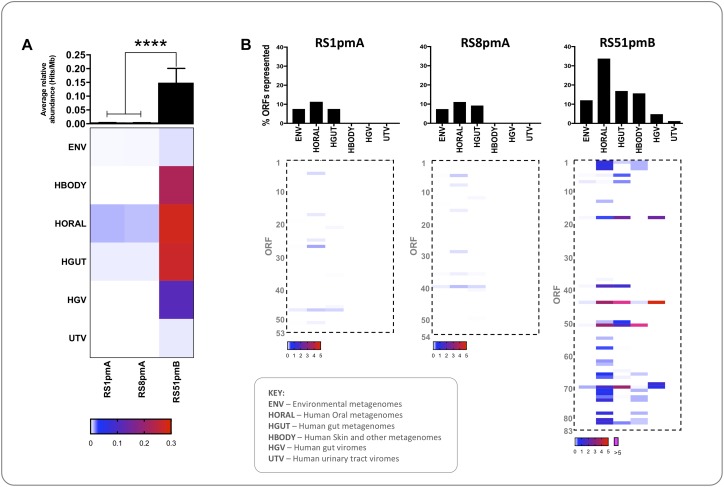
Ecogenomic profiling of *P. mirabilis* phage encoded functions. The representation of functions encoded by *P. mirabilis* phages in a range of microbial habitats was investigated by calculating the relative abundance of similar sequences in 809 metagenomic data sets. Valid hits from tBlastn searches with *P. mirabilis* phage sequences (≥35% identity, ≥50% query coverage, ≤1e^– 5^) were used to calculate the average relative abundance of ORF homologs in each dataset (expressed as Hits/Mb). **(A)** The heatmap shows average relative abundance of ORFs from each phage in metagenomes or viromes from various habitats. Rows represent datasets grouped by habitat, and columns represent individual phage. The intensity of shading in cells indicate cumulative relative abundance of ORFs from a given phage in metagenomes from the corresponding habitat (according to the scale shown). The associated histogram shows average relative abundance of phage ORFs across all datasets. **(B)** Heatmaps show cumulative relative abundance of individual ORFs from each phage (Rows) in metagenomes from various habitats (Columns), and shading of cells indicates relative abundance according to the associated scale. Associated histograms show the proportion of ORFs in each phage with homologs detected in various habitats represented by metagenomes surveyed. ^∗∗∗∗^*P* < 0.0001 (Kruskal-Wallis test with Dunn’s correction), error bars show standard error of the mean. RS1pmA is used to represent the previously described group of phage RS1pmA, RS1pmB, and RS3pmA (Nzakizwanayo et al., 2016). ENV, whole community metagenomes with non-host associated environmental origin (*n* = 16); HGUT, HORAL, and HBODY, whole community metagenomes from the human gut microbiome, the human oral cavity, or various human body sites (primarily external) (*n* = 285, 295, and 181, respectively). HGV, viral metagenomes from the human gut (*n* = 12). UTV, viral metagenomes from the human urinary tract (*n* = 20). Details of datasets used can be found in Supplementary Table S2.

To gain further insight into the broader ecological associations of these *P. mirabilis* phages, we also investigated the relative representation of sequences similar to *P. mirabilis* phage encoded ORFs in other bacterial ecosystems (Figure 5). No phage analyzed exhibited notable representation in metagenomes derived from human urinary tract viromes or environmental datasets, but associations with other human-derived habitats were observed (Figure 5A). As with the other analyses conducted here, RS1pmA and RS8pmA exhibited similar profiles, which were markedly different to those obtained for RS51pmB (Figures 5A,B). ORFs with similarity to those encoded by RS1pmA and RS8pmA were only poorly represented in the metagenomic datasets examined, with no statistically significant representation in any habitat, but with most homologs identified in datasets from the human oral cavity (Figure 5B).

In comparison, sequences similar to RS51pmB encoded ORFs showed significantly greater representation in metagenomic datasets than RS1pmA and RS8pmA, and a particular association with human-associated microbial or viral communities from the oral cavity, gastrointestinal tract and other body sites (Figure 5). In the case of RS51pmB, a wider range of ORFs was found to be generally well represented in the metagenomic datasets examined, with greatest representation in human oral microbiomes (ORFs 44 and 51), human gut microbiomes and viromes (ORFs 18, 44, 51, and 70), and human body associated datasets (ORF 51). These ORFs were predicted to encode a large terminase subunit (ORF 51); methyltransferases (ORFs 18 and 70) and a structural protein (ORF 44) (Figure 5B and Supplementary Data S1).

## Discussion

The potential for phages to control CAUTI, and in particular biofilm formation on urinary catheters, has previously been demonstrated using various models of infection (Curtin and Donlan, 2006; Carson et al., 2010; Fu et al., 2010; Lehman and Donlan, 2015; Melo et al., 2016; Nzakizwanayo et al., 2016). Phages offer a number of advantages over conventional antimicrobials for the control of biofilm-associated infections, including mechanisms evolved to penetrate biofilms and access bacterial hosts normally protected by the matrix of extracellular polymeric substances (Sutherland et al., 2004; Lu and Collins, 2007). Phages are also not generally affected by a host species susceptibility to conventional antimicrobial agents, and may therefore also contribute to efforts to control antimicrobial resistance and address this global challenge.

This makes phages potentially well suited for controlling infections where biofilm formation is an important feature, and situations in which conventional antimicrobial agents often perform poorly (Sutherland et al., 2004; Lu and Collins, 2007; Stickler, 2014; Melo et al., 2016; Nzakizwanayo et al., 2016). There is also the potential to use phage in synergy with conventional antibiotic treatments, which may have considerable benefits in terms of enhanceing treatment efficacy, as well as curtailing the emergence of antibiotic resistant strains. It is also worth noting that phages isolated in this and our previous study were all recovered from sewage samples, using clinical isolates of *P. mirabilis* as the host species (Nzakizwanayo et al., 2016). This further highlights that phages with potential therapeutic value can be easily recovered from such sources, and it is likely that sewage and a wastewater harbor diverse phage populations useful for a range of applications.

The majority of phages characterized in this study produced plaques with halos, as described previously for phages shown to be effective in controlling *P. mirabilis* crystalline biofilm formation (Nzakizwanayo et al., 2016). The most common explanation for plaques with halos is the presence of PD activity in phages. The expression of PD enzymes is believed to facilitate phage infection of host cells through degradation of exterior capsules, or by providing access to biofilm associated cells through the disruption of the encasing exopolymeric matrix (Sutherland et al., 2004; Lu and Collins, 2007). This feature appeared to be most apparent among the members of the *Podoviridae* family characterized in this study (RS1pmA, RS1pmB, RS3pmA, and RS8pmA), and these observations were congruent with characteristics of *P. mirabilis* phages PM16 and PM75 to which podophage examined in this study showed notable similarity in terms of gene content (Morozova et al., 2016, 2018). The potential importance of PD activity in the control of biofilm formation has perhaps been most clearly demonstrated by Lu and Collins, via the engineering of phage T7 (Lu and Collins, 2007). In this study, T7 phages augmented to express PD activity were significantly enhanced in their ability to disperse established biofilms as compared to wild-type counterparts (Lu and Collins, 2007).

However, no ORFs encoded by phages characterized in this study were identified as encoding potential PD activity (based on BlastP and Conserved Domain analyses); but it is possible that these phages encode novel PD enzymes not currently well represented in sequence databases. Alternatively, halos generated by these phages may be the result of other processes, such as lysis inhibition, where superinfection of cells leads to delays in cell lysis and a zone of partially lysed cells around plaques that can also manifest as a translucent halo (Hershey, 1946). Nevertheless, the putative PD activity of these phages, and confirmation of the presence or absence of this activity, is likely to be important in further studies of their potential to disrupt *P. mirabilis* biofilms. PD activity may also have more direct pharmaceutical potential in its own right, in terms of developing novel anti-biofilm agents.

Other features of these phage genomes may also hold similar pharmaceutical potential in the context of developing novel antimicrobial agents, such as ORFs predicted to be involved in host cell lysis (Endersen et al., 2015; Czaplewski et al., 2016). Of particular interest are the putative endolysins encoded by RS1pmA and RS8pmA, which are involved in degradation of the host cell wall to facilitate phage release during the final stages of lytic replication. Although these enzymes have been found to be highly effective against Gram-positive bacteria when applied as exogenous preparations, the use of endolysins to control Gram-negative bacteria has been hampered by the presence of the outer lipopolysaccharide (LPS) membrane in these species, which prevents access to the peptidoglycan layer and blocks the anti-microbial action of these enzymes (Zimmer et al., 2002; Donovan et al., 2006; Doehn et al., 2013; Briers et al., 2014; Briers and Lavigne, 2015; Oliveira et al., 2016). The identification and characterization of putative endolysins from phages infecting Gram-negative bacteria may provide important insights and biological raw materials to address this challenge. There is already precedence for the identification of exogenously active Gram-negative endolysins from phages infecting *Acinetobacter baumanii*, and their potential use as alternatives to antibiotics (Lai et al., 2011; Lood et al., 2015; Oliveira et al., 2016), as well as for the engineering of these enzymes to improve spectrum of activity (Briers et al., 2014; Yang et al., 2014; Briers and Lavigne, 2015).

The genetic analysis of phages characterized here also provided further insight into their performance in previous evaluations of phage-mediated control of catheter blockage (Nzakizwanayo et al., 2016), and their suitability with regard to further development of phage therapy applications. We previously demonstrated that a cocktail of RS1pmA, RS1pmB, and RS3pmA could provide a significant increase in time taken for catheters to block during simulations of established CAUTI (Nzakizwanayo et al., 2016). When applied to models simulating early colonization of the catheterized urinary tract, these phages were able to eradicate infection and completely prevent catheter blockage, whereas under conditions simulating established infection they were only able to delay blockage (Nzakizwanayo et al., 2016).

Characterization of the corresponding phage genomes in this study now demonstrates these phages vary only by point mutations in their genomes. This supports the hypothesis that the eventual therapeutic failure during simulations of established infection may have been due to development of resistance (Nzakizwanayo et al., 2016), made possible by a lack of diversity in the cocktail used. This further highlights the utility of phage genome characterization in developing cocktails containing diverse and distinct phage types, to offset development of resistance in phage therapy applications. It should also be noted that the activity of phages we characterized here have been evaluated against only a small number of *P. mirabilis* isolates to date. Recent serotyping studies indicated a high diversity among UTI isolates with no predominant serotype (Kaca et al., 2011). Analysis of phage activity against a broader panel of well-defined *P. mirabilis* strains will also be important to the development of phage therapies, and *P. mirabilis* strain diversity may present additional challenges in this regard.

The characterization of phage genome sequences conducted here also highlighted how more expansive characterization can illuminate important facets of phage-host relations, and broader ecological affiliations that may be relevant to development of diverse, and effective combinations for phage therapy. Comparison of ORFs encoded by these phages, with ORFs in other phage genomes, showed most phages (RS1pmA, RS1pmB, RS3pmA, and RS8pmA) had a strong association with other available *P. mirabilis* phage genomes. This further highlighted the potential novelty of RS51pmB, in which most ORFS were not similar to those in any other phage genomes evaluated, and a clear divergence from other *P. mirabilis* phages was evident.

To further illuminate broader phage-host relationships relevant to the development of phage therapies, tetranucleotide usage profiling was employed, which highlighted the potential for extending host range to other problematic species involved in CAUTI. The hypothesis underlying the tetranucleotide approach is that co-evolution of host and phage leads to the development of tetranucleotide usage profiles in phage genomes that are similar to those found in the chromosomes of long-term bacterial hosts (Pride et al., 2006; Ogilvie et al., 2012, 2013). As such, phages that have more recently adapted to infect a particular host should theoretically exhibit less conformity in this genome-signature, and may exhibit tetranucleotide usage profiles more aligned to previous or alternative host species, providing insight into host-phage evolutionary relationships (Pride et al., 2006; Ogilvie et al., 2012, 2013).

This analysis revealed that RS51pmB was much less closely related to *P. mirabilis* than the other phages analyzed, and instead was most closely associated with a range of other members of the *Morganellaceae* family. The genome signature-based prediction was subsequently supported by host range assays which demonstrated the capacity for RS51pmB to infect and replicate within *M. morganii*, albeit relatively inefficiently compared with replication in *P. mirabilis* and only when used at high titres. Taken together, these data indicated that, in evolutionary terms, RS51pmB may have relatively recently adapted to infect and replicate within *P. mirabilis*, and the tetranucleotide genome signature of this phage has not yet acclimated to the *P. mirabilis* host.

Although a potential transition of this phage from *P. mirabilis* to *M. morganii* cannot be fully excluded, the clustering of RS51pmB with *M. morganii* based on the tetranucleotide genome signature, and the apparent ability to more readily replicate in *P. mirabilis*, is more compatible with a transition from *Morganella* to *Proteus*. This also raises the possibility of utilizing such phage as the basis to more directly develop derivatives with an extended spectrum of activity, for example using passage-based methods such as the Appelmans technique (Appelmans, 1921) and a wider range of *M. morganii* clinical isolates. This approach has already been utilized in the host range extension of *P. mirabilis* phages for development of broad-spectrum cocktails (Lehman and Donlan, 2015). Nevertheless, it should also be noted that while tetranucleotide profiling has provided evidence that RS51pmB has a past relationship with *M. morganii*, the host-range assays undertaken in this study remain limited in scope overall. More expansive studies with a greater range of bacterial strains should now be undertaken to further evaluate the host-phage associations we observe based on genome-signatures, and test the robustness of the inferred evolutionary relationships. In particular, it will be important to understand how consistent and reliable such inferences are across different phage-host groups, datasets, and taxonomic scales.

Characterization of phage diversity, and the ecological success of different phages or phage encoded genes through ecogenomic profiling, also has the potential to provide insights into phages that are useful in biotechnological applications (Ogilvie et al., 2012, 2013, 2018). This information may help guide the selection of phages for inclusion in cocktails, or those with properties useful in other biotechnological applications, by facilitating identification of ecologically diverse phages and those that have particular associations with given habitats (Ogilvie et al., 2012, 2013, 2018). Notably, in the ecogenomic analyses conducted here, there was a lack of affiliation of any phage characterized with the human urinary viromes analyzed, despite the dominance of phage associated sequences in these datasets, and the diagnosis of UTI in 50% of the individuals from which they were derived (Santiago-Rodriguez et al., 2015). While this may initially seem contradictory, it is unlikely that *P. mirabilis* would constitute a member of the normal human urinary microbiome, and none of the culture-positive samples from which viromes were derived were found to contain *P. mirabilis* (Santiago-Rodriguez et al., 2015).

Conversely, ecogenomic profiling reinforced the novelty of RS51pmB observed in comparisons with other phage genomes, but also provided a greater understanding of the broader ecological associations of these phages. A distinct affiliation with the human alimentary microbiome (oral and gut) was observed for RS51pmB, and to a lesser extent RS1pmA and RS8pmA, in keeping with *P. mirabilis* being a common member of the human gut microbiome and the isolation of these phages from sewage. The human gut-associated ecogenomic profiles of RS51pmB may also highlight the possible value of this phage in other biotechnological applications, such as in the development of virome-based microbial source tracking tools for monitoring water quality (Adriaenssens and Brister, 2017; Ogilvie et al., 2018). The gene-by-gene nature of this analysis may also be relevant to bioprospecting studies seeking to exploit specific attributes or functions encoded by phages, where representation or activity within a particular habit may be desirable. In this context, the recovery of these phage from sewage reinforces the potential for wastewater treatment facilities to also provide access to a wealth of biological raw materials encoded by phages. However, it should also be noted that ecogenomic studies are currently restricted by the available metagenomic datasets, which represent only a relatively small sample size for many habitats, and leave many microbial ecosystems unrepresented (Ogilvie et al., 2012, 2013, 2018). Further research will be required to fully understand how phage ecological profiles may best be used to inform the development of phage therapy or exploit phage for other applications.

Overall, the genetic characterization of phages considered in this study, including evaluation of wider host-phage, ecological, and evolutionary relationships, highlight the potential for these broader genomic and ecogenomic assessments to assist in identifying phage with the most potential for pharmaceutical or biotechnological exploitation, or suitability for use in phage therapy. It is also clear that a range of easily accessible “waste” materials, such as sewage and wastewater, constitute useful sources of novel and biotechnologically relevant phages. Given the current rise in antimicrobial resistance and urgent need to address this global challenge, it seems likely that alternatives or supplements to conventional small molecule antimicrobials will become an important feature of strategies to address this problem (Zimmer et al., 2002). Phages are well suited to form an important part of this solution, particularly with regard to biofilm-associated infections, and approaches to help identify the most promising phages for pharmaceutical or biotechnological exploitation will be vital to the realization of more effective phage-based products.

Currently, the selection of phages to be used for the development of therapeutic applications is understandably driven by phenotypic characterization and activity against target pathogens, with genetic characterization often a secondary activity primarily used to screen for undesirable genes such as toxins. However, the generation of phage genome sequences is now accessible and affordable, and the increasing range of tools available for analyzing these data mean this aspect of phage characterization should now be viewed as a primary process in the selection of candidate phage for the development of therapeutic products, or other biotechnological applications. Although next generation sequencing technologies generating short reads (such as Illumina platform sequencing) can pose challenges in the correct assembly of some phage genomes, such approaches can still provide useful information regarding phage gene content and broader evolutionary relationships. However, technologies such as single molecule nanopore sequencing can potentially generate genome length reads, and we demonstrate here some of their potential to address many of the current limitations of next generation sequencing for phage genome analysis, related to read length. Furthermore, platforms such as the Oxford Nanopore MinION require no specialist genomics facilities or support to operate, offering the possibility for genome characterization of newly isolated phages to become a much more routine aspect of phage characterization.

## Data Availability

The datasets generated for this study can be found in the GenBank under the accession numbers MG575418, MG575419, and MG575421.

## Author Contributions

BJ and DA conceived the study. All authors designed and conducted the experiments, analyzed the data, and edited the manuscript. BJ, LO, and JN wrote the manuscript.

## Conflict of Interest Statement

The authors declare that the research was conducted in the absence of any commercial or financial relationships that could be construed as a potential conflict of interest.

## Footnotes

1 https://github.com/rrwick/Porechop
