# Supplementary material for: Genomic and Ecogenomic Characterization of Proteus mirabilis Bacteriophages
Source: Front Microbiol. 2019 Aug 6;10:1783. doi: 10.3389/fmicb.2019.01783 (PMC6691071; doi:10.3389/fmicb.2019.01783)
Supplement: Supplementary file 1 [file Data_Sheet_1.pdf]

Supplementary Information for Alves et al.,

"Genomic and ecogenomic characterisation of *Proteus mirabilis* Bacteriophages"

Table S1: Bacterial host strains and phage characterised in this study.

Species	Strain	Phage characterised [a]					Origin [c]
		RS1pmA[b]	RS1pmB[b]	RS3pmA[b]	RS8pmA	RS51pmB	
Proteus mirabilis	RS1	+	+	+	-	-	Clinical isolate from CAUTI
Proteus mirabilis	RS3	+	+	+	-	-	Clinical isolate from CAUTI
Proteus mirabilis	RS8	-	-	-	+	-	Clinical isolate from CAUTI
Proteus mirabilis	RS10	-	-	-	-	-	Clinical isolate from CAUTI
Proteus mirabilis	RS51	-	-	-	-	+	Clinical isolate from CAUTI
Morganella morganii	NCIMB 232	-	-	-	-	+/-	From NCIMB culture collection
Providencia stuartii	DSM2250	-	-	-	-	-	From DSM culture collection
Providencia rustigannii	DSM2520	-	-	-	-	-	From DSM culture collection
Providencia retgeri	DSM4542	-	-	-	-	-	From DSM culture collection
Serratia marcescens	NCTC 1377	-	-	-	-	-	From NCTC Culture Collection
Enterobacter cloacae	NCTC 8155	-	-	-	-	-	From NCTC Culture Collection
Klebsiella pneumnoniae	B4193	-	-	-	-	-	Clinical isolate

a **+** = Host used to isolate phage; **+** Susceptible to phage; **-** not susceptible to phage; **+/-** replication only observed when high titres of phage used. Host range assays were performed in triplicate at phage titres ranging from ~10-10^7 PFU

b RS1pmA, RS1pmB, RS3pmA are indicated to be strains of the same phage.

c Origin of bacterial hosts used. CAUTI = Catheter Associated Urinary Tract Infection.

Table S2: Overview of datasets and sequences utilised.

Dataset type	Habitat/Sequence type	Source[1]	Reference/comment
Viral Metagenomes	Human Gut *	NCBI SRA	(Reyes et al., 2010)
	Urinary Tract	Author Correspondence	(Santiago-Rodriguez et al., 2015)
Whole Community Metagenomes	Human Gut (MetaHit) – Danish, Spanish	EMBL	(Qin et al., 2010) http://www.bork.embl.de/~arumugam/Qin_et_al_2010/
	Human Gut – Japanese	CAMERA	(Kurokawa et al., 2007)
	Human Gut – American	CAMERA	(Gill et al., 2006)
	NIH Human Microbiome Project (All body sites)	NIH	(Nelson et al., 2010) http://hmpdacc.org/. Accessed Feb 2014
	Termite Gut	CAMERA	(Warnecke et al., 2007)
	Global Ocean Sampling Expedition	CAMERA	(Yooseph et al., 2007; Rusch et al., 2007)
	Waseca County Farm Soil	CAMERA	(Tringe et al., 2005)
	Acid Mine Drainage	CAMERA	(Tyson etal., 2004)
	Washington Lake	CAMERA	(Kalyuzhnaya et al., 2008)
	Marine Metagenome	CAMERA	Gordon and Betty Moore Foundation Marine Microbiology Initiative. Sequenced at the Broad Institute: http://www.broadinstitute.org/annotation/viral/Phage/Home.html
	Whale Fall	CAMERA	(Tringe et al., 2005)

[1] Datasets and genome sequences utilised in this project were obtained from a range of publically accessible repositories:

CAMERA (Sun et al., 2011): Community Cyberinfrastructure for Advanced Microbial Ecology Research and Analysis.

CAMERA Homepage: https://portal.camera.calit2.net/gridsphere/gridsphere. Datasets now available from Cyverse iMicrobe: http://imicrobe.us/

NCBI: National Centre for Biotechnology Information (http://www.ncbi.nlm.nih.gov).

NCBI SRA: Pyrosequencing reads generated from virus-like particles by Reyes et al., (2010) were obtained from the NCBI short read archive, project SRA012183 (http://www.ncbi.nlm.nih.gov/sra).

EMBL: Metagenomes comprising the MetaHIT dataset (Qin et al., 2008) were obtained from the European Molecular Biology Laboratory database *via* the link provided in the table.

HMP: NIH Human Microbiome Project **(**http://hmpdacc.org/)

***** Assembled viral metagenomes utilised for these datasets were generated using CAMERA workflows (Sun et al., 2011), as described in Ogilvie et al., (2012).

Table S3: Summary of ORFs detected in mature viral particles from phage characterised.

Phage	ORF [a]	Unique peptides	Coverage (%)	Predicted function [a]
RS1pmA	34	10	22.20	Head-tail connector
	36	28	63.06	Capsid protein
	39	3	16.76	Tail protein
	41	25	30.98	Tail protein
	42	4	22.43	Internal virion associated protein
	43	31	29.99	Polynucleotide kinase
	44	34	25.66	Internal virion associated protein
	45	10	21.45	Tail fibre protein
	48	8	67.88	Virion associated protein
	52	14	26.21	Tail protein
RS8pmA	37	11	18.66	Tail Connector
	39	26	64.26	Capsid protein
	42	4	17.3	Tail protein
	43	29	31.62	Tail protein
	44	5	24.77	Virion associated protein
	45	30	34.78	Polynucleotide kinase
	46	42	32.20	Internal core protein
	47	11	25.14	Tail fibre protein
	50	5	27.14	Virion associated protein
	54	23	31.10	Virion associated protein
RS51pmB	63	1	7.01	Unknown phage associated
	64	3	8.1	Capsid protein

a - See **Dataset S1** for further details of ORFs and **Figure 2** for physical maps of phage genomes. Shading of predicted ORF functions reflects broad functional groupings used in Figure 2 and Dataset S1: **Green** – Structure and Packaging; Orange - Replication and Regulation; Blue – Unknown function but homologues in other phage genomes; Grey - Unknown function, not detected in other phage genomes.

References

1. Reyes, A., Haynes, M., Hanson, N., Angly, F. E., Heath, A. C., Rohwer, F., *et al.* (2010). Viruses in the faecal microbiota of monozygotic twins and their mothers. *Nature* 466, 334–338. doi: 10.1038/nature09199

2. Santiago-Rodriguez, T. M., Ly, M., Bonilla, N., and Pride, D. T. (2015).The human urine virome in association with urinary tract infections. *Front Microbiol.* 6, 14. doi: 10.3389/fmicb.2015.00014

3. Qin, J., Li, R., Raes, J., Arumugam, M., Burgdorf, K. S., Manichanh, C., *et al.* (2010). A human gut microbial gene catalogue established by metagenomic sequencing: Commentary. *Natur*e 464, 59-65. doi: 10.1038/nature08821

4. Kurokawa, K., Itoh, T., Kuwahara, T., Oshima, K., Toh, H., Toyoda, A., *et al.* (2007). Comparative metagenomics revealed commonly enriched gene sets in human gut microbiomes. *DNA Res.* 14, 169–181. doi: 10.1093/dnares/dsm018

5. Gill, S. R., Pop, M., Deboy, R. T., Eckburg, P. B., Turnbaugh, P. J., Samuel, B. S., *et al.* (2006). Metagenomic analysis of the human distal gut microbiome. *Science* 312, 1355–1359. doi: 10.1126/science.1124234

6. Nelson, K. E., Weinstock, G. M., Highlander, S. K., Worley, K. C., Creasy, H. H., Wortman, J. R., *et al.*(2010). A Catalog of Reference Genomes from the Human Microbiome. *Science* 328, 994–999. doi: 10.1126/science.1183605

7. Warnecke, F., Luginbühl ,P., Ivanova, N., Ghassemian, M., Richardson, T. H., Stege, J. T., *et al.* (2007). Metagenomic and functional analysis of hindgut microbiota of a wood-feeding higher termite. *Natur*e 450, 560–565. doi: 10.1038/nature06269

8. Yooseph, S., Sutton, G., Rusch, D. B., Halpern, A. L., Williamson, S. J., Remington, K., *et al.* (2007).The Sorcerer II Global Ocean Sampling Expedition: Expanding the Universe of Protein Families. *PLoS Biol.* 5,e16. doi: 10.1371/journal.pbio.0050016

9. Rusch, D. B., Halpern, A. L., Sutton, G., Heidelberg, K. B., Williamson, S., Yooseph, S., *et al.* (2007). The Sorcerer II Global Ocean Sampling Expedition: Northwest Atlantic through Eastern Tropical Pacific. *PLoS Biol.* 5, e77. doi: 10.1371/journal.pbio.0050077

10. Tringe, S. G., von Mering, C., Kobayashi, A., Salamov, A. A., Chen, K., Chang, H. W., *et al.*(2005). Comparative metagenomics of microbial communities. *Science* 308, 554–557. doi: 10.1126/science.1107851

11. Tyson, G. W., Chapman, J., Hugenholtz, P., Allen, E. E., Ram, R. J., Richardson, P. M., *et al.* (2004). Community structure and metabolism through reconstruction of microbial genomes from the environment. *Nature* 428, 37–43. doi: 10.1038/nature02340

12. Kalyuzhnaya, M. G., Lapidus, A., Ivanova, N., Copeland, A. C., McHardy, A. C., Szeto, E., *et al.* (2008). High-resolution metagenomics targets specific functional types in complex microbial communities. *Nat Biotechnol.* 26, 1029–1934. doi: 10.1038/nbt.1488

13. Sun, S., Chen, J., Li, W., Altintas, I., Lin, A., Peltier, S., *et al.*(2011). Community cyberinfrastructure for Advanced Microbial Ecology Research and Analysis: the CAMERA resource. *Nucleic Acids Res.* 39, D546-D551. doi: 10.1093/nar/gkq1102

14. Edgar, Robert C. (2004). MUSCLE: multiple sequence alignment with high accuracy and high

throughput, *Nucleic Acids Res.* 32(5), 1792-97. doi: 10.1093/nar/gkh340

15. Ogilvie, L. A., Caplin, J., Dedi, C., Diston, D., Cheek, E., Bowler, L., *et al*. (2012). Comparative (Meta)genomic Analysis and Ecological Profiling of Human Gut-Specific Bacteriophage B124-14. *PLoS ONE* 7, e35053. doi: 10.1371/journal.pone.0035053

Supple Fig 1

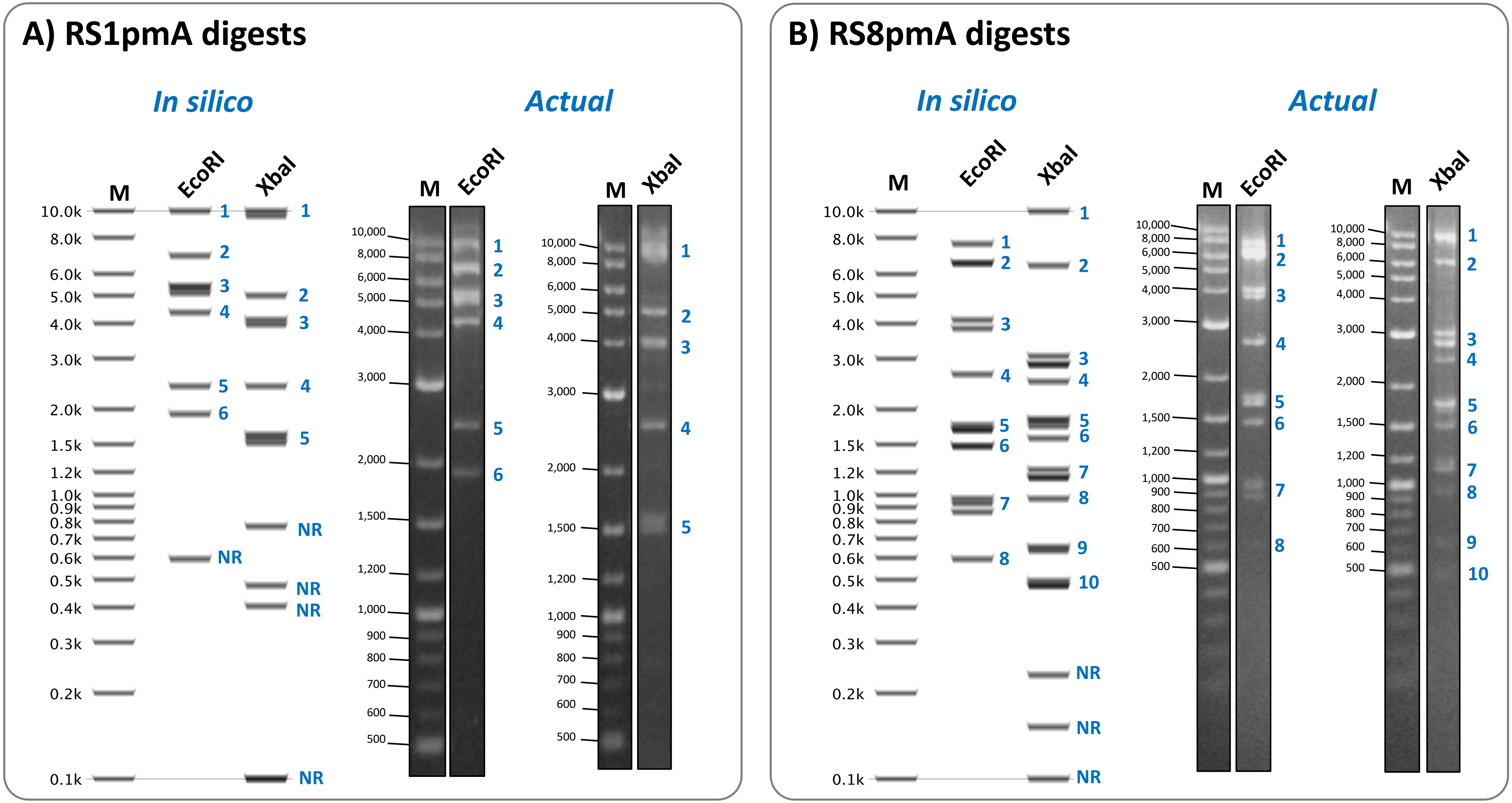

Supplementary Figure 1 : Restriction digest of RS1pmA and RS8pmA genomes: For both parts: Numerals - indicate relative position**.** To confirm the physical genome structure indicated by minION sequencing and correction of illumina assemblies, phage were subject to digest with restriction endonucleases *EcoRI* and *XbaI*, and profiles compared to predicted patterns nanopore-corrected genome sequences (Fig 2). **A)** Shows results of *in silico* and actual digests of RS1pmA. **B)** Shows results of *in silico* and actual digests of RS8pmA ns of main features on *in silico* digests and actual restriction profiles. **NR** – denotes restriction fragments predicted by in silico digests but below the size that were clearly resolved on the agarose gels of actual digests. For virtual gels, fragments positioned inline with the 0.1kb size standard represent all fragments below this size and expected sizes are provided in parentheses. **M** – DNA size standard (NEB 2-log ladder).

Supple Fig 2

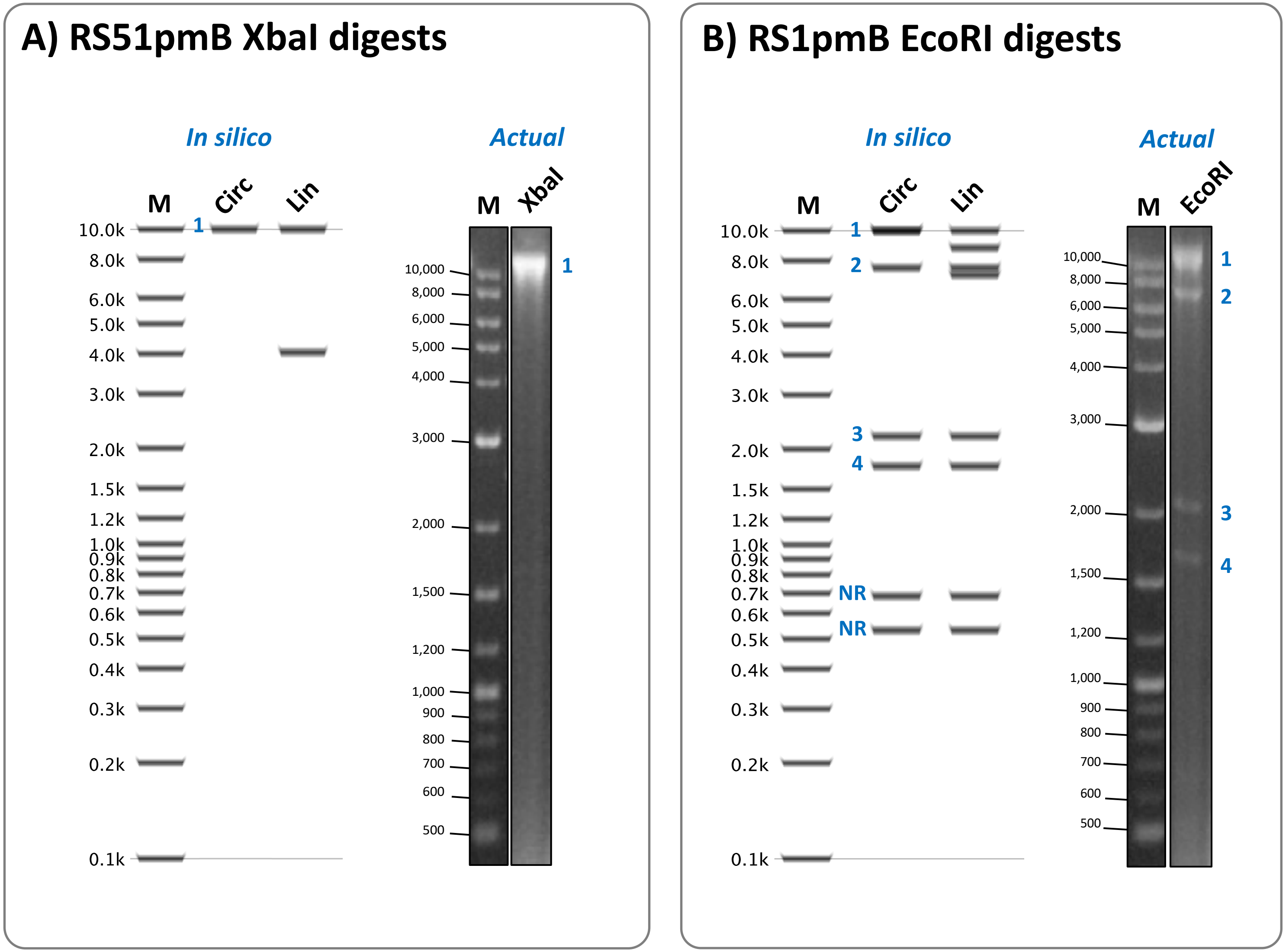

Supplementary Figure 2: Restriction digest of RS51pmB genome. To evaluate the physical genome structure indicated by illumina and minION sequencing RS51pmB was subject to digest with restriction endonucleases EcoRI and XbaI, and profiles compared to predicted patterns of the RS51pmB genome in both circular (**Circ**) and Linear (**Lin**) forms (Fig 2). **A)** Shows results of *in silico* and actual digests with *XbaI*. **B)** Shows results of *in silico* and actual digests with *EcoRI*. For both parts: ***Numerals -*** indicate relative positions of main features on *in silico* digests corresponding to actual restriction profiles. ***NR*** – denotes restriction fragments predicted by *in silico* digests but not clearly resolved on the agarose gels of actual digests. **M** – DNA size standard (NEB 2-log ladder).

Supple Fig 3

RS1pmA, RS1pmB, RS3pmA

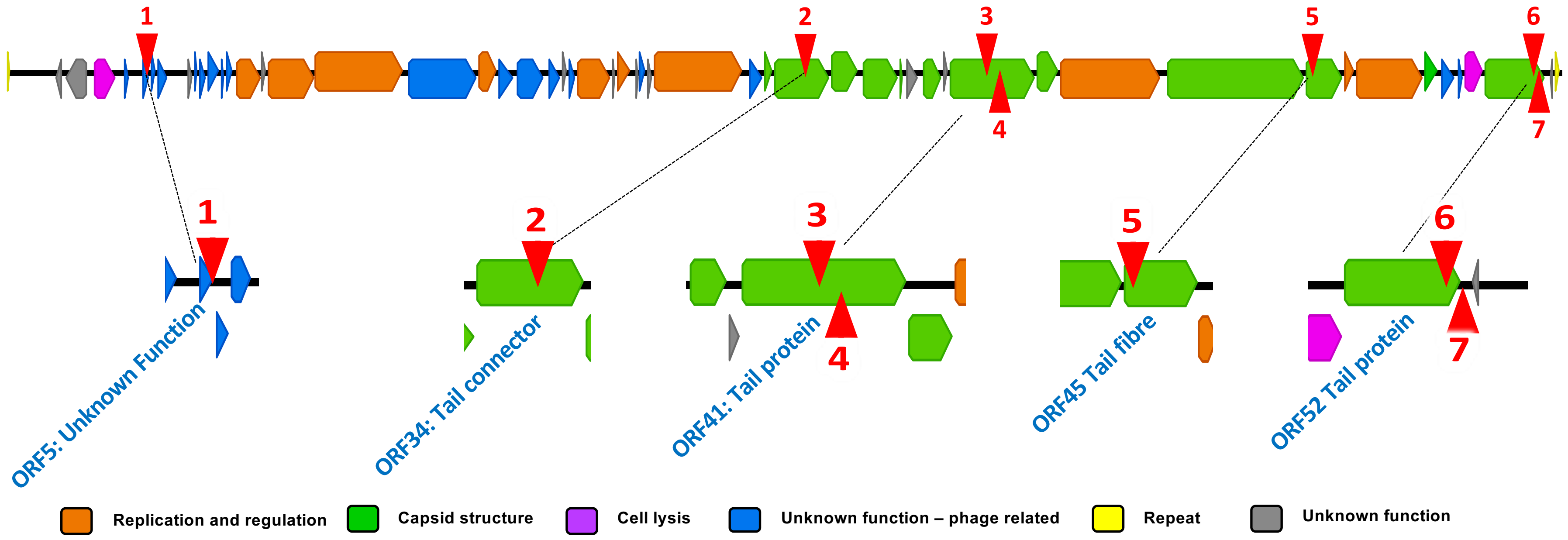

SNP	Phage	ORF (See also Data S1)	Effect
1	RS3pmB	ORF3 Unknown function (phage related)	Silent
2	RS1pmA	ORF32 Tail connector protein	Cys>Arg
3	RS3pmB	ORF39 Tail protein	Silent
4	RS1pmA	ORF39 Tail protein	Thr>Ile
5	RS1pmA	ORF43 Tail fibre prtoein	Arg>His
6	RS1pmB	ORF50 Tail associated protein	Glu>Lys
7	RS1pmA	ORF50 Tail associated protein	Asp>Gly

Supplementary Figure 3: Genomes of P. mirabilis RS1pmA, RS1pmB and RS3pmA vary only by point mutations. Comparison of genome sequences from RS1pmA, RS1pmB and RS3pmA revealed that these differ only by single nucleotide polymorphisms (SNPs). The physical genome map shows the position of SNPs in phage genomes, and the associated table indicates phage in which each SNP was identified and the predicted effect on amino acid sequences encoded by relevant ORFs.

Supple Fig 4

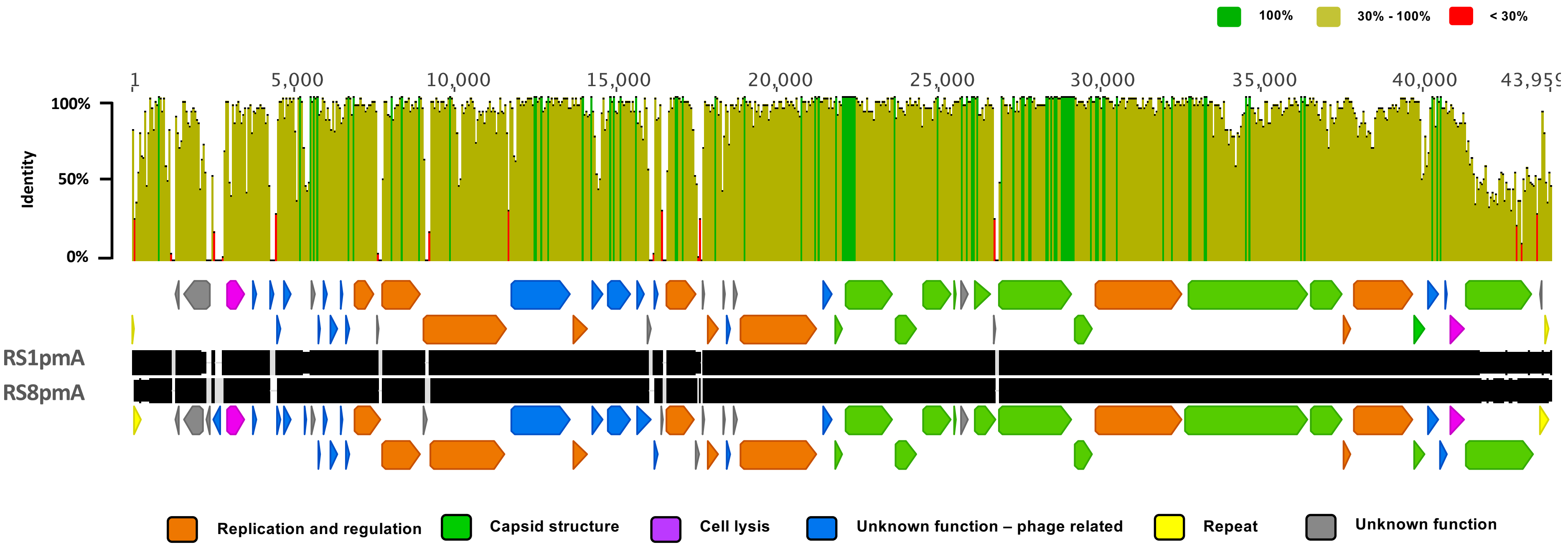

Supplementary Figure 4: Comparison of RS8pmA and RS1pmA genomes. Pairwise alignments RS1pmA and RS8pmA genome sequences were generated using MUSCLE (14), implemented and visualised using Geneious 9.1.8. Upper chart shows mean pairwise identity between sequences, where height of bars indicates % identity to the consensus of both phage, and shading indicates broad identity ranges as denoted by the associated key. Block arrows denote ORFs and shading corresponds to broad functional categories as assigned in Fig 2 and described in the associated key. Thickness of associated horizontal bars show areas of genomes that align and gaps in alignments.

Supple Fig 5

A

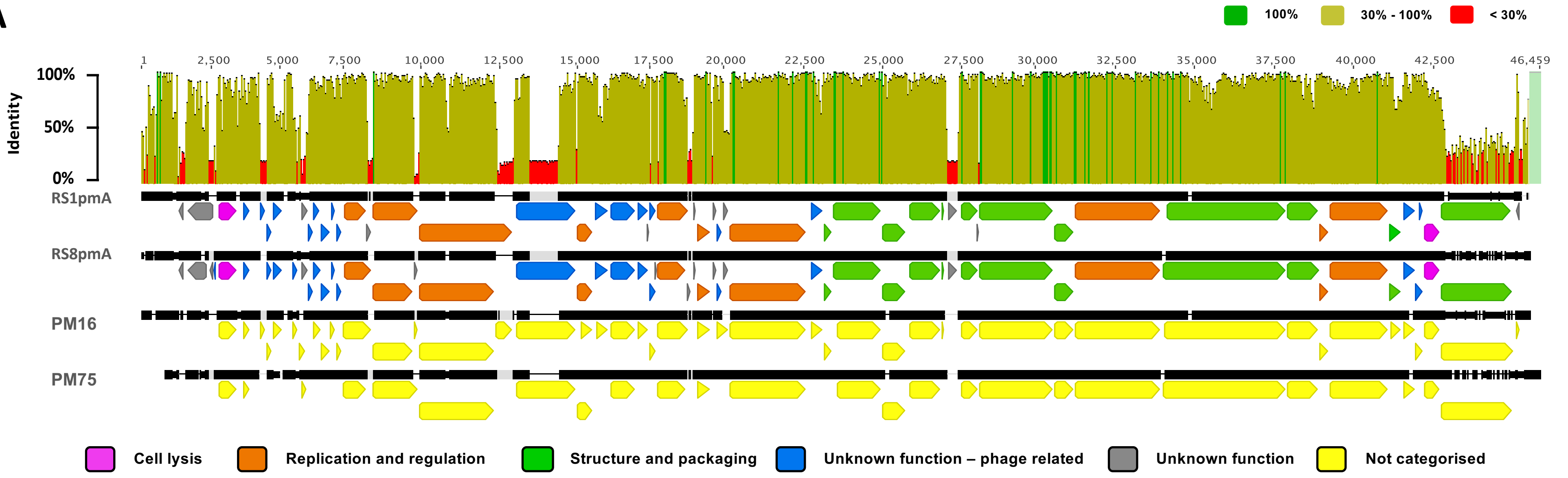

B

	RS1pmA	RS8pmA	PM16	PM75
RS1pmA	100%			
RS8pmA	83.44%	100%		
PM16	82.05%	80.14%	100%	
PM75	79.82%	80.46%	82.62%	100%

Supplementary Figure 5: Comparison of RS1pmA and RS8pmA genomes with other closely related P. mirabilis phage. Due to the similarity between RS1pmA and RS8pmA with other *Proteus* phage genomes in ORF by ORF comparisons (Fig 3), the whole genomes sequences of these phage were compared with phages PM16 and PM75 by pairwise alignments. Genomes were aligned using MUSCLE (14), implemented Geneious 9.1.8. **A)** Upper chart shows mean pairwise identity between sequences relative to the majority consensus sequence derived from all genomes, where height of bars indicates % identity and shading indicates broad identity ranges as denoted by the associated key. Block arrows represent ORFs and show gene synteny between phage genomes over alignments, with shading corresponding to broad functional categories as assigned in Fig 2 and described in the associated key. Thickness of associated horizontal bars show areas of genomes that align and gaps in alignments. **B)** Matrix showing overall pairwise similarity from alignments between each phage genome, expressed as % nucleotide identity.
